# The Effect of Vanadate, Phosphate, Fluoride Compounds on the Aqueous Corrosion of Magnesium Alloy AZ31 in Dilute Chloride Solutions

**DOI:** 10.3390/ma13061325

**Published:** 2020-03-14

**Authors:** Zhiyuan Feng, Jichao Li, Zi Yang, Rudolph Buchheit

**Affiliations:** 1 Department of Chemical and Materials Engineering, University of Kentucky, Lexington, KY 40502, USA; Rudolph.Buchheit@uky.edu; 2 Department of Materials Science and Engineering, The Ohio State University, Columbus, OH 43210, USA; li.2037@buckeyemail.osu.edu (J.L.); yang.684@osu.edu (Z.Y.)

**Keywords:** light alloys, magnesium, corrosion, vanadate, phosphate, fluoride, inhibition, conversion coating

## Abstract

The anodic polarization response of magnesium alloy AZ31 was first characterized during exposure to aerated 0.1 M NaCl solutions with millimolar additions of NaVO_3_, Na_3_PO_4_, Na_2_HPO_4_, NaF and various pairings to assess their ability to inhibit corrosion kinetics and retard localized corrosion. Each of the candidate inhibitors reduced the corrosion rate of the alloy to some degree. A Na_3_PO_4_–NaVO_3_ pair produced a good inhibiting effect decreasing the corrosion rate to about 10^−7^ A/cm^2^, which was two orders of magnitude lower than the uninhibited control case. A Bliss Independence assessment indicated that this inhibitor pair acted synergistically. A Na_2_HPO_4_–NaVO_3_ pair reduced the corrosion rate to 10^−6^ A/cm^2^ but was not assessed to be acting synergistically. The NaVO_3_–NaF pair did not reduce the corrosion rate significantly compared to the control case and was an antagonistic pairing. SEM imaging showed film formation due to exposure, which appears to be the origin of the observed inhibition. The resistance to localized corrosion was assessed as the difference in the breakdown potential and the corrosion potential, with larger values indicating a lower probability of localized corrosion during free corrosion exposures. The effects of the inhibitors on this characteristic were mixed, but each of the inhibitor pairs yielded potential differences in excess of 100 mV. A conceptual conversion coating process based on a mixture of vanadate and phosphate compounds were demonstrated. A fluoride-bearing formulation produced coatings whose total impedance was increased by a factor of two compared to an uncoated control. A fluoride-free formulation produced coatings whose corrosion resistance was increased by more than a factor of three.

## 1. Introduction

The high strength-to-density ratio makes magnesium alloys attractive for use in applications where light-weighting is an important performance attribute. As a result, the use of magnesium is increasing in automotive, aircraft and other manufacturing sectors [1,2]. Unlike the other light metals—aluminum and titanium—the passivity of magnesium is not robust except at very high pH. For this reason, magnesium alloys experience comparatively high corrosion rates for humid or condensing atmospheric service conditions [1,3,4], and the long-term durability of magnesium alloys in engineered products depends on protective coating systems.

Multi-component coating systems for magnesium alloys comprising anodized or conversion coating foundation layers overlaid with corrosion-inhibiting primers and protective paint layers are effective in protecting magnesium [3,5,6,7,8,9,10]. The chromate conversion coating, strontium chromate primer barrier, andcoating paint system used for the protection of aluminum alloys is also effective for protecting magnesium alloys [11,12]. Coating systems of this type provide opportunities for the storage and release of corrosion-inhibiting agents to provide an active component of corrosion protection that supplements the barrier protection provided by the coating system.

The toxic nature of chromate and the growing restrictions placed on the use of chromate-bearing coating systems is well documented and prompts the search for chromate-free protective systems for magnesium alloys, just as it has for aluminum alloys [13]. In fact, some countries have banned chromate coatings for all but the most essential applications [14].

Because the surface conversion of magnesium alloys is an essential part of coating systems for magnesium alloys, and due to restrictions on the use of chromate-based conversion processes, protective non-chromate conversion coating materials and processes are needed. For example, surface conversion chemistries based on phosphate, cerium, fluoride, vanadate, selenite and molybdate have demonstrated a protective effect on magnesium [15,16,17,18,19,20,21,22,23].

Inhibitor screening approaches are useful for identifying chemical agents that may be the basis for surface conversion processes, and these approaches have been pursued in this study. The use of vanadate, phosphate, and fluoride compounds singly and in combination has been examined. These compounds were selected as a possible basis for a non-chromate conversion coating process.

Vanadate has been shown to be a good inhibitor of the 2024-T3 aluminum alloy [24,25,26], and a vanadate conversion coating process that demonstrates a self-healing response has been reported for that alloy [19]. Prior studies of vanadate inhibition on Mg alloys indicate that vanadate alone does not provide a sufficiently potent inhibiting effect on Mg by itself, and one or more companion inhibitors may be needed for technological applications [20,21,27]. Vanadium is not a cancer-causing chemical in mammals, but may do damage to the respiratory system under high concentration exposure [22]. In animal tests, deaths occurred in rabbits exposed to 114 mg vanadium/m^3^ for 1 h [28]. One candidate companion is phosphate or a hydrophosphate species. Insoluble phosphate films with protective characteristics will form on Mg alloys [29,30,31,32]. Fluoride is another candidate companion species. Like phosphate, fluoride forms an insoluble and protective MgF_2_ film on Mg [3,33].

This work focuses on the combined inhibiting effect and resulting conceptual conversion coating process based on the use of the three species mentioned above. AZ31 was used as an exemplar magnesium alloy for this study. Corrosion rate measurements were conducted using anodic polarization and electrochemical impedance spectroscopy (EIS), and the surface morphology was characterized by SEM. Within the inhibitor assessment portion of this work, various combinations were evaluated to determine if any of the combinations acted synergistically. Finally, a conceptual conversion coating process was devised based on the evaluation of inhibitor combination efficacy. Conversion coatings were applied, and an initial screening assessment of the resulting corrosion protection during short-term exposure of converted surfaces to dilute chloride solutions was carried out.

## 2. Materials and Methods

### 2.1. Inhibitor Characterization

A commercial AZ31 Mg alloy sheet (2.5-3.5 Al, 0.7-1.3 Zn, and 0.2 Mn on a wt.% basis) was used for all experiments. The alloy sheet was cut into 40 × 40 mm pieces, and polished using silicon carbide (SiC) paper with ethanol as lubricant, starting from 600 and finishing with 1200 grit. The polished samples were ultrasonically cleaned in ethanol. Cleaned samples were then dried using a hot air gun.

NaVO_3_ (Acros Organics 96%), Na_3_PO_4_ (Alfa Aesar 99%), Na_2_HPO_4_ (Alfa Aesar 98%), and NaF (Acros Organics 99%) were used as candidate inhibitors in the study. The solution base was 0.1 M NaCl with one or two of the inhibitors, which were added in different concentrations. Some of the inhibitors had the capacity to buffer the solution, and control experiments were carried out at pH 5.0, 7.7 and 9.2 to account for this possible effect. The inhibitor content of each experiment is described in Table 1.

The extent of inhibition was characterized using anodic polarization, which was carried out using a lab-made traditional three-electrode vertical corrosion cell (sample was facing up). A saturated calomel electrode (SCE) was used as the reference electrode and platinum mesh was used as the counter electrode. Measurements were made after 10, 30 and 60 min open-circuit exposures allowing chloride and the inhibitors present to interact with the surface, as dictated by their respective activities in solution.

During potentiodynamic polarization, the potential was scanned at a rate of 0.5 mV/s, and the initial potential was −50 mV vs. the open circuit potential (OCP). After anodic polarization, samples were dried and the corrosion morphology was examined using scanning electron microscopy. Samples were imaged using accelerating voltages ranging from 10 to 20 kV, which produced images that sampled the depth of surfaces by several hundred nanometers to a few micrometers.

### 2.2. Conversion Coating Characterization

Two conversion coating baths were prepared to form conversion coatings on AZ31 samples. The first was an aqueous mixture of 10 mM NaVO_3_, 10 mM Na_3_PO_4_, 10 mM Na_2_HPO_4_ and 1 mM NaF. The second bath had an identical composition, but excluded NaF. AZ31B coupons were rinsed with deionized water and immersed in the conversion bath for 1, 5, or 10 min. After coating, all samples were aged in air for 24 h before further testing. Corrosion protection was assessed using electrochemical impedance spectroscopy (EIS), which was carried out in 0.1 M NaCl after a 30 min exposure to the solution. EIS measurements were made in a cell formed from a 5 cm high plastic tube with a 5.5 cm^2^ cross-sectional area that was affixed to the sample. EIS data were collected at a rate of seven points per decade over a frequency range of 100 kHz to 10 mHz.

## 3. Results and Discussion

### 3.1. Characterization of AZ31 Response in Chloride-Only Solutions

**Figure 1:**
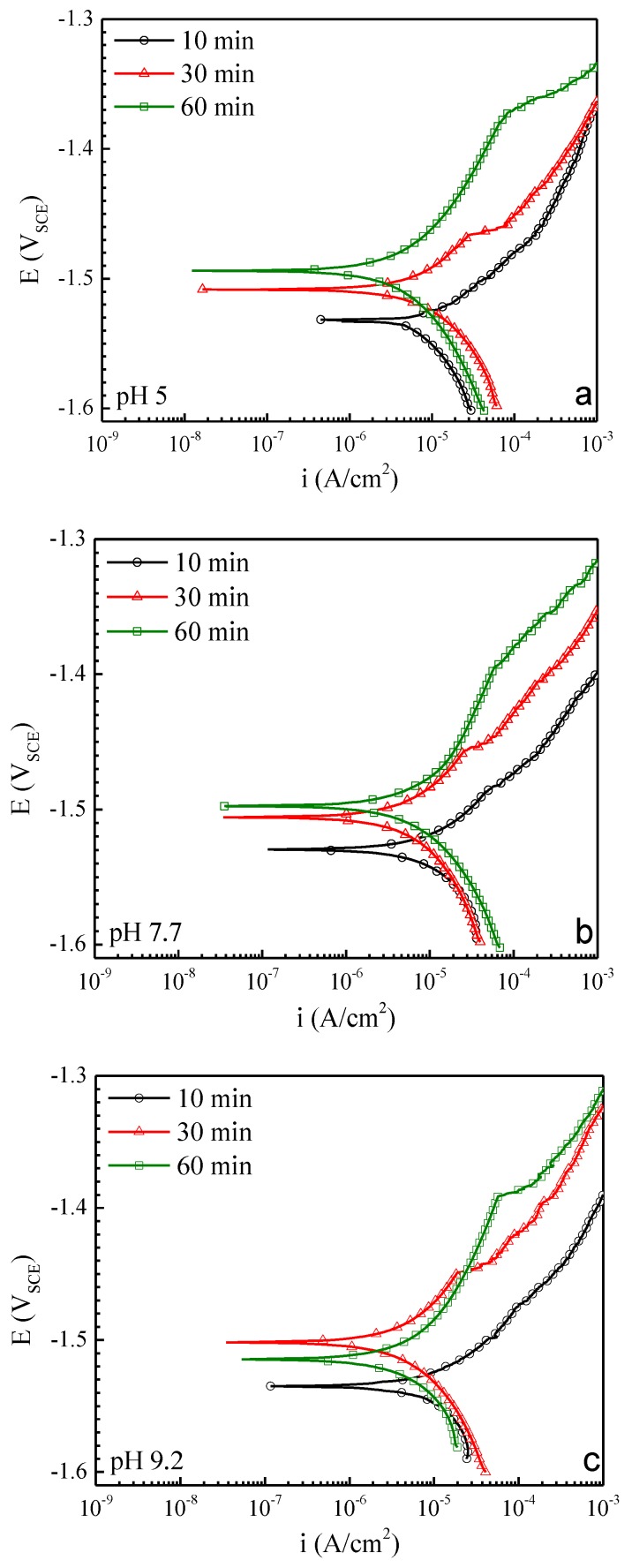
Anodic polarization curves for AZ31 in 0.1 M NaCl at pH 5 (**a**), pH 7.7 (**b**), and pH 9.2 (**c**).

Figure 1 shows the anodic polarization curves for AZ31 in 0.1 M NaCl at pH 5.0, 7.7 and 9.2 after various immersion times (10, 30 and 60 min). These curves, which represent the uninhibited control response in the study, were collected, starting about 50mV below the corrosion potential, where the rate of water reduction is about 0.1 mA/cm^2^. Under these conditions, the test solution becomes alkaline due the high rate of water reduction and the absence of appreciable Mg^2+^ hydrolysis. This occurs independent of initial solution pH and, for that reason, the polarization responses are similar across the pH range examined. There is also a similarity in the anodic polarization response over the 10 to 60 min pre-exposure time frame examined.

In general, the corrosion potential is observed to increase by 30 to 40 mV, and the breakdown potential is observed to increase by about 100 mV as exposure time increases to 60 min. The corrosion rate, as determined by Tafel analysis, is in the 5 to 15 µA/cm^2^ range, and the rate remains steady or decreases slightly as the exposure time increases.

These changes in the polarization response indicate a time-dependent passivation process that produces a marginal increase in resistance to localized corrosion. The solution used in these experiments was open to the air and CO_2_ was dissolved into the solution. Over the pH range examined, CO_2_ speciates to bicarbonate, HCO_3_^−^ and carbonate, CO_3_^2−^. These species can react with magnesium and aluminum hydroxides at a neutral to moderately alkaline pH to produce a mixed Mg–Al hydroxycarbonate that confers a slight protective effect [34,35].

### 3.2. Inhibitor Characterization of AZ31 Polarization Response in Chloride-Only Solutions in Single and Mixed Inhibitors

**Figure 2:**
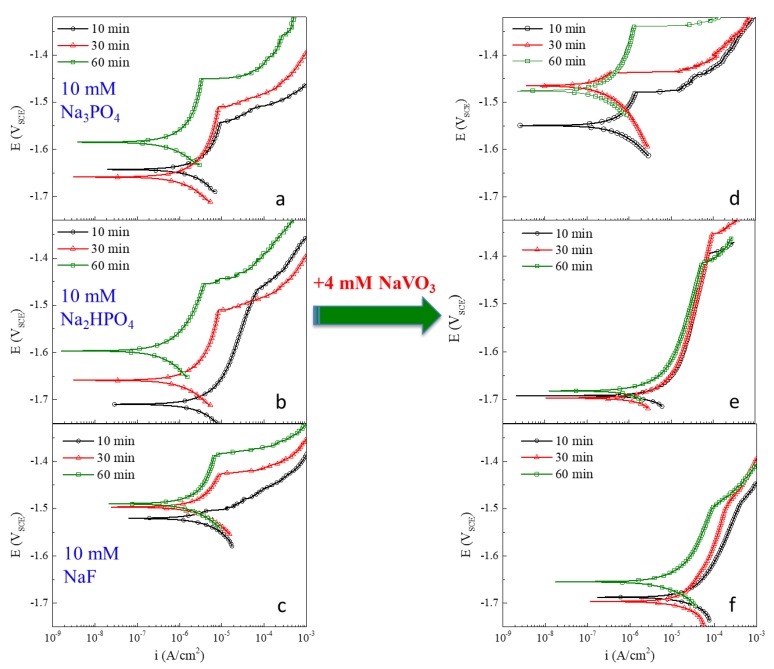
Polarization curves for AZ31 in 0.1 M NaCl with (**a**) 10 mM Na_3_PO_4_, (**b**) 10 mM Na_2_HPO_4_, (**c**) 10 mM NaF, (**d**) 4 mM NaVO_3_ plus 10 mM Na_3_PO_4_, (**e**) 4 mM NaVO_3_ plus 10 mM Na_2_HPO_4_, and (**f**) 4 mM NaVO_3_ plus 10 mM NaF. Three polarization curves are shown for each instance after 10, 30 and 60 min of open circuit exposure to solution.

Figure 2a–f show the representative anodic potentiodynamic polarization response for AZ31 for each inhibitor solution in the presence of 0.1 M NaCl. All experiments were replicated three times. The polarization response of each experiments was highly reproduceable. Corrosion current density values were determined by extrapolation of the linear portion of the log-based cathodic polarization curves to the intersection with corrosion potential values. Figure 2a–c show the response in the presence of 10 mM Na_3_PO_4_, Na_2_HPO_4_, and NaF, respectively. Figure 2d–f show the response in those same solutions with the addition of 4 mM NaVO_3_. All figures are plotted on the same scale to facilitate comparison.

Na_3_PO_4_: the addition of phosphate, Na_3_PO_4_, alone to 0.1M NaCl (Figure 2a) has the effect of decreasing the corrosion rate, but also decreasing the breakdown potential and the corrosion potential of AZ31 relative to the control response in the chloride-only solution shown in Figure 1. The inhibiting action of phosphate on AZ31 is slow. The largest change in polarization response occurs between the 30 and 60 min pre-exposure observations. Phosphates are film formers on magnesium alloys and film formation appears to slow anodic and possibly cathodic kinetics in these experiments. The corrosion rate is reduced by as much as one order of magnitude in the presence of 10 mM phosphate. The resistance to passive film breakdown for AZ31 under free corrosion conditions, as characterized by the difference between the breakdown potential and the corrosion potential, is increased in the presence of Na_3_PO_4_, but the absolute breakdown potential is reduced by about 50 mV compared to the control case in the chloride-only solution.

Na_3_PO_4_ plus NaVO_3_: the addition of 4 mM NaVO_3_ to the 10 mM Na_3_PO_4_ solution has a significant effect on the polarization response, which indicates that additional corrosion protection is conferred to AZ31. The observed corrosion rates fall below 1 µA/cm^2^, which is nearly an order of magnitude less than the phosphate-only rate, and nearly two orders of magnitude less than the rates measured in the chloride-only solution.

Compared to AZ31 exposed to the combined inhibitor solution for only 10 min, the dissolution kinetics are ennobled significantly by exposure for 30 or 60 min. In addition, the breakdown potential is elevated more by the addition of vanadate to phosphate than any other case examined in this study. This suggests prompt inhibiting action by vanadate that continues to act to increase resistance to localized corrosion over time. After 60 min pre-exposure, the absolute breakdown potential is slightly above that measured in the chloride-only solution.

Na_2_HPO_4_: the addition of 10 mM Na_2_HPO_4_ to the chloride solution produces effects on the polarization response for AZ31 that indicate inhibition. There is a strong decrease in the corrosion potential to about −1.7 V_SCE_ after 10 min exposure. The corrosion potential gradually drifts in the positive direction and increases −1.6 V_SCE_ after 60 min of exposure. This is still 100 mV more negative that the corrosion potential observed in the chloride-only solution. As the corrosion potential increases, the corrosion rate decreases to a few tenths of a µA/cm^2^ after 60 min. There is a well-articulated breakdown potential at all exposure times, but no steady increase or decrease in the potential at which breakdown occurs. The range of breakdown potentials falls in the range of −1.45 to −1.50 V_SCE_, which is 100 mV below the most noble breakdown potentials observed in the chloride-only solutions. There is a significant change in overall anodic kinetics over time, indicating the slower and steadier action of the inhibitor; similar to the inhibiting action observed with the addition of Na_3_PO_4_. Overall, given the decrease in corrosion rate and the large separation in breakdown and corrosion potential, NaHPO_4_ imparts slowly accumulating corrosion inhibition on AZ31.

Na_2_HPO_4_ plus NaVO_3_: the addition of 4 mM NaVO_3_ to 10 mM Na_2_HPO_4_ produces a polarization response, indicating a mixed effect on corrosion inhibition for AZ31. Hydrophosphate alone has the effect of depressing the corrosion potential and gradually decreasing the corrosion rate as exposure time increases. In moderately alkaline solutions, vanadate alone has the effect of increasing the corrosion potential, increasing the breakdown potential slightly, and decreasing the corrosion rate. [21] When mixed together under the conditions of these experiments, hydrophsophate and vanadate combine to strongly decrease the corrosion potential, increase the breakdown potential and decrease slightly the corrosion rate of AZ31 compared to chloride-only exposures.

NaF: sodium fluoride is often used as an activating agent in metal finishing processes. However, with magnesium alloys, it can lead to the formation of insoluble and protective MgF_2_ by a reaction with soluble Mg^2+^. Figure 2c shows that the addition of 10 mM NaF to a 0.1 M NaCl solution does not affect the polarization response significantly. The characteristic potentials, the corrosion rate, and the time-dependent evolution of the polarization response are all remarkably similar to those shown in the polarization curves of Figure 1.

NaF plus NaVO_3_: the combination of 4 mM NaVO_3_ and 10 mM NaF shifts the corrosion potential in the negative direction to nearly −1.7V_SCE_. There is a slight increase in corrosion rate with the dissolution rate in the passive region, increasing through 100 µA/cm^2^, before breakdown is reached at about −1.5V_SCE_. Although there is a 200 mV separation in breakdown and corrosion potential suggesting a low risk of localized corrosion under free corrosion conditions; the breakdown potentials in this case are about 100 mV lower than those observed in the chloride-only solution after a 60 min exposure.

**Figure 3:**
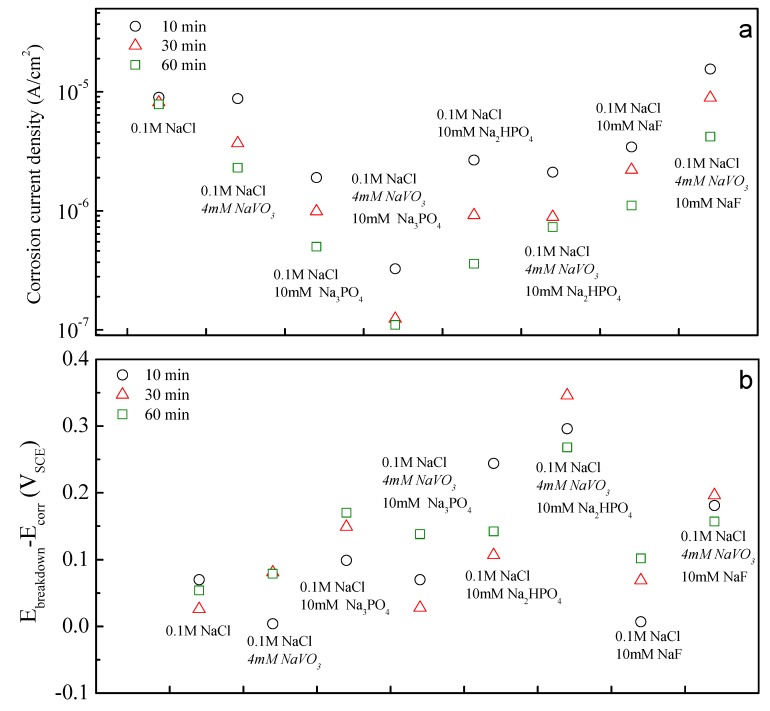
A summary of (**a**) corrosion current density and (**b**) E_breakdown_-E_corr_ that were examined in this study.

Inhibitor efficacy: the average corrosion current densities and the difference in the breakdown potential were aggregated and are plotted in Figure 3a,b for each inhibitor and inhibitor combination examined in this study. These data show that there are several inhibitors and inhibitor combinations that reduce corrosion rate by one order of magnitude or more. These include Na_3_PO_4_, Na_2_HPO_4_, NaF and the Na_3_PO_4_–NaVO_3_ pair. The Na_3_PO_4_–NaVO_3_ pair is noteworthy among these. This pair reduced the corrosion rate by two orders of magnitude compared to the corrosion rate in the chloride-only control case. For all the inhibitors examined, the corrosion rate was observed to decrease with increasing exposure time. The inhibiting action, as assessed by the change in corrosion rate from 10 to 60 min, appeared to manifest somewhat more quickly when vanadate was present and somewhat more slowly when only phosphate or hydrophosphate was present. This was attributed to the rapid absorption-based inhibition attributable to vanadates and the slower precipitation-based inhibition attributable to phosphates [36].

The magnitude of the difference between the breakdown potential and the corrosion potential is a qualitative measure of the resistance to localized corrosion under free corrosion conditions with larger values, indicating smaller probabilities of localized attack. Among the single inhibitors examined, Na_3_PO_4_ and Na_2_HPO_4_ demonstrated a difference of more than 100 mV. Among the inhibitor pairs, Na_3_PO_4_–NaVO_3_, Na_2_HPO_4_–NaVO_3_, and NaF–NaVO_3_ each demonstrated a difference of more than 100 mV.

While vanadates, phosphates and fluorides are all good inhibitors for magnesium alloys, these results show that some combinations of inhibitors can provide greatly enhanced corrosion inhibition.

### 3.3. Assessing the Strength of Inhibitor Pair Interactions

In the case of paired corrosion inhibitors, it is possible to assess and classify their combined effect as additive, synergistic (when the effect is more than additive), or antagonistic (when the effect is less than additive) [37,38].

Inspection of the corrosion rate or E_breakdown_-E_OCP_ shows that all the inhibitor pairs inhibited corrosion of AZ31; however, a more detailed assessment was conducted using the Bliss Independence method to determine if any of the inhibitor combinations were acting synergistically [37,38]. In this method, a synergism parameter (S) is derived to assess the effect [39,40,41]:

(1) $$S=\frac{1-\left(\eta_{1}+\eta_{2}-\eta_{1}\eta_{2}\right)}{1-\eta_{1+2}},$$

where η_1_, η_2_ and η_1+2_ represent the quantitative characteristics of corrosion inhibition noted in this study, corrosion rate and the difference in breakdown potential and corrosion potential, respectively. If S > 1, the effect of the inhibitor pair is synergistic, if S < 1 the effect is antagonistic, and if S = 1 the effect is additive. The magnitude of S above and below one is a measure of the degree of synergy or antagonism.

The values of corrosion rate and potential difference were normalized for use in Equation 1, as follows:

(2) $$\eta_{i}=\frac{i_{{\mathrm{corr}}_{0}}-i_{\mathrm{corr}}}{i_{{\mathrm{corr}}_{0}}};$$

(3) $$\eta_{\mathrm{pit}}=\frac{E_{\mathrm{diff}}-E_{{\mathrm{diff}}_{0}}}{E_{{\mathrm{diff}}_{0}}}.$$

In these expressions, i_corr_ is the corrosion current density in the presence of an inhibitor or inhibitor pair. i_corr0_ represents the corrosion current density in 0.1 M NaCl only. Similarly, E_diff_ is the value of E_breakdown_–E_OCP_ in the presence of an inhibitor or inhibitor pair. E_diff0_ represents the value of E_breakdown_–E_OCP_ in 0.1 M NaCl only.

A “mixing effect” was also determined for the inhibitor pairs. The mixing effect was positive when the effect of the inhibitor pair was greater than the effect of either of the individual inhibitors, and negative when the effect of the pair was less than either of the individual inhibitors. The mixing effect was determined using the normalized values of the corrosion rate and the difference in the breakdown and corrosion potentials. A positive mixing effect satisfies the condition:

(4) $$\eta_{1}\eta_{2}>{\;\eta }_{1},{\;\eta }_{2},$$

while a negative mixing effect satisfies the condition:

(5) $$\eta_{1}\eta_{2}<{\;\eta }_{1},{\;\eta }_{2}.$$

The synergy parameter, S, and the mixing effect for the effect of inhibitor combinations and exposure times on AZ31 corrosion rate is shown in Table 2. For the Na_3_PO_4_–NaVO_3_ inhibitor pair, the mixing effects are all positive and the S parameters are all larger than one with some being much greater than one, and a synergistic effect is indicated. For the Na_2_HPO_4_–NaVO_3_ inhibitor pair, the mixing effect is positive at 10 and 30 min of pre-exposure, but not for 60 min. The S parameter is 1.24 at 10 min but drops below one thereafter. In this case, corrosion inhibition is indicated for a short exposure time with an effect on corrosion rate that is slightly more than additive, but protection is not persistent over time, with an accumulating antagonistic effect. A strong antagonistic effect is indicated for the NaF–NaVO_3_ inhibitor pair. The mixing effect is negative for all pre-exposure times, and the S parameter is persistently less than one.

Table 3 shows the mixing effect and S parameters determined from the difference in breakdown potential and corrosion potential. In this case, the Na_3_PO4–NaVO_3_ inhibitor pair demonstrates a persistently negative mixing effect and an antagonistic effect on the potential difference characteristic. These results do not indicate a reinforced resistance to localized corrosion. The Na_2_HPO_4_–NaVO_3_ inhibitor pair tends to decrease the corrosion potential and increase the breakdown potential slightly, suggesting an increase in resistance to spontaneous localized corrosion. The pair demonstrates a positive mixing effect, and the S parameter indicates that this pair acts synergistically on the potential difference characteristic. Although sodium phosphate and sodium hydrogen phosphate are closely related chemical compounds, they interact with vanadate in very different ways, leading to different inhibition profiles.

The effect of the NaF–NaVO_3_ on the magnitude of the difference between the breakdown potential and the corrosion potential is synergistic with a positive mixing effect. Fluoride is generally used as an activator in conversion coating formulations and it is a film-former for Mg alloys. This action, combined with the adsorption inhibition of vanadate, appears to produce the effect. The S parameters calculated for this pair do not vary much over the pre-exposure times examined, suggesting that the inhibiting action of the pair is prompt and persistent.

For the inhibitor pairs examined in this study, a consideration of the mixing effect and the assessment of synergy, additivity or antagonism does not change the rank ordering of inhibition effectiveness that might be made based on an inspection of the corrosion rate shown in Figure 3a. However, it does discriminate among results to show where synergistic interactions are occurring within a set of larger results, most of which indicate a positive effect in reducing the corrosion rate. The overall indications point to a strong and persistent synergistic effect with the Na_3_PO_4_–NaVO_3_ inhibitor pair. Even though there is an antagonistic effect on the difference in the breakdown and corrosion potential, that difference stands at about 100 mV after 60 min of exposure to the solution, suggesting that the tendency for localized corrosion under free corrosion conditions is still low in the presence of these two inhibitors.

### 3.4. Post-Exposure Surface Morphology

**Figure 4:**
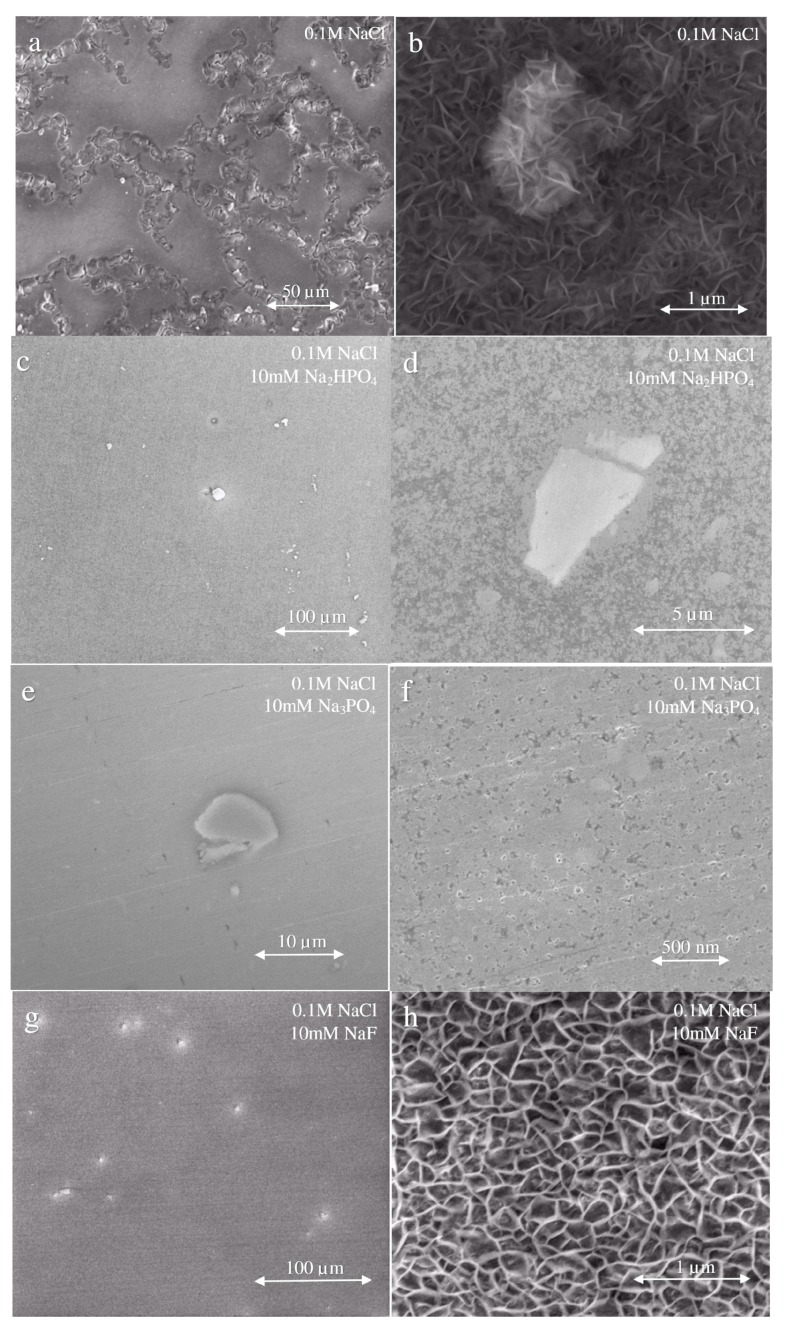

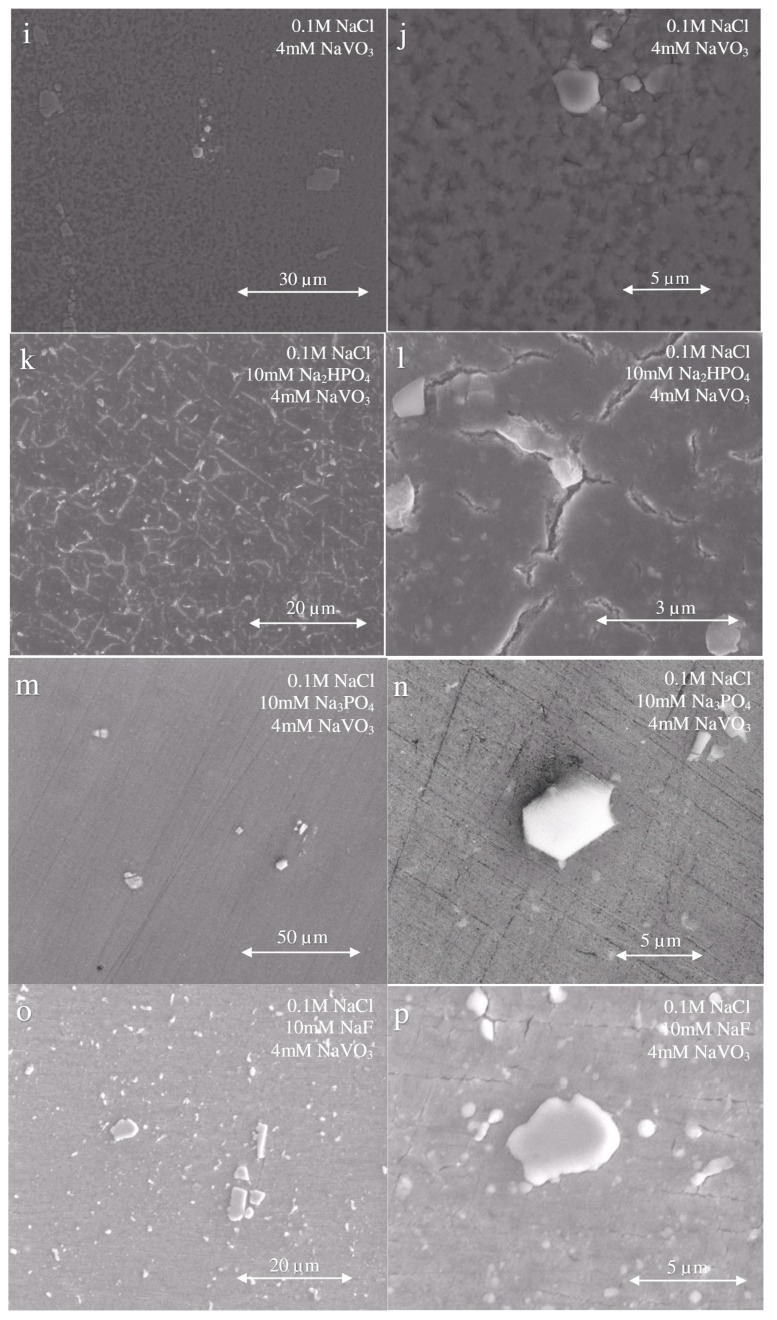
Scanning electron micrographs of AZ31 after 1 h exposures to inhibitor and inhibitor pairs at room temperature.

After exposure to the various inhibitor mixtures, surfaces were examined by scanning electron microscopy. Figure 4 shows the resulting surface morphologies. Figure 4a,b is the morphology of AZ31 after 1 h immersion in uninhibited 0.1 M NaCl. The matrix of the alloy is heavily corroded and covered with porous corrosion products (Figure 4a). At the high magnification (Figure 4b), a filamentous morphology is resolved. The corrosion product on the surface mainly contains a combination of MgO and Mg(OH)_2_ [34,42,43]. When Na_2_HPO_4_ is added into 0.1 M NaCl (Figure 4c,d), a protective film is formed on the surface and the second phase particles are not attacked, which can be seen at a high magnification (Figure 4d). A similar surface morphology is presented when 10 mM Na_3_PO_4_ is added into 0.1 M NaCl (Figure 4e,f), but film coverage is more extensive than for Na_2_HPO_4_ (Figure 4f). A distinctive surface morphology results from exposure to NaF-bearing solutions (Figure 4g). These exposures result in a highly structured film whose morphology resembles that of a double-layer hydroxide compound (Figure 4h) [44]. Exposure to vanadate-bearing solutions results in a continuous and featureless film across the alloy surface (Figure 4i,j). The absence of shrinkage cracks suggest that the film produced is very thin. A somewhat thicker film is produced during exposure to 0.1 M NaCl with 4 mM NaVO_3_, and 10 mM Na_2_HPO_4_ (Figure 4k,l). A uniform film is produced like the one associated with the presence of vanadate in solution. However, shrinkage cracks are observed, suggesting that a thicker film has formed than in the case of vanadate-only exposure. In Figure 4m,n, the film is formed under exposure of the combination of 0.1 M NaCl, 4 mM NaVO_3_, and 10 mM Na_3_PO_4_. The surface is uniform and apparently dense, even at high magnification (Figure 4n). Figure 4o,p are from surfaces exposed to 0.1 M NaCl, 4 mM NaVO_3_ and 10 mM NaF. In Figure 4o, some white spots that represent the second phase particles are distributed on the surface. At high magnification (Figure 4p), some slight shrinkage cracking can be observed on the surface.

### 3.5. Conceptual Conversion Coatings for AZ31

Based on the results from electrochemical testing and examination of film formation in those experiments, two different chemistries of coating baths were formulated, and coatings were formed on AZ31 surfaces by an immersion process. The first was a solution comprising 0.01 M NaVO_3_, 0.01 M Na_2_HPO_4_, 0.01 M Na_3_PO_4_, and 0.001 M NaF. The second was 0.01 M NaVO_3_, 0.01 M Na_2_HPO_4_, 0.01 M Na_3_PO_4_, with the main difference in the formulations being the presence or absence of 1 mM fluoride. 

AZ31 coupons were cleaned by wiping with ethanol and were immersed into the coating for 1, 5, or 10 min. After coating, the color of the coated surface was pale yellow, which is an indication of pentavalent vanadium in the coating. The coatings were allowed to age in air for 24 h prior to any further handling. Coated samples were immersed directly into aerated 0.1M NaCl for 30 min and then EIS spectra were collected.

**Figure 5:**
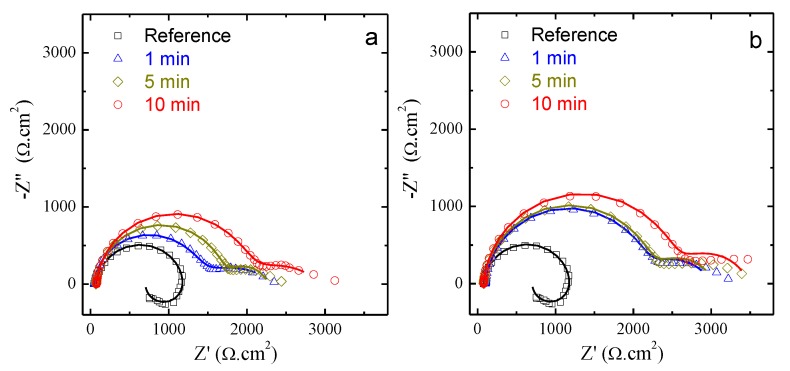
Impedance spectra of conversion coated AZ31 coated for 1, 5 or 10 min then aged in air for 24 h. Bare AZ31 is shown for comparison. (**a**) Coating bath formulation: 0.01 M NaVO_3_, 0.01 M Na_2_HPO_4_, 0.01 M Na_3_PO_4_, and 0.001 M NaF; (**b**) coating bath formulation: 0.01 M NaVO_3_, 0.01 M Na_2_HPO_4_ and 0.01 M Na_3_PO_4_.

Figure 5a,b show the Nyquist plots of AZ31 coupons coated in the fluoride-containing and fluoride-free coating baths, respectively. The impedance of uncoated AZ31 was also measured for comparison.

The impedance spectra from coated AZ31 samples is shown in Figure 5a,b. The spectrum from the uncoated sample presents a capacitive loop and an inductive loop at a low frequency. Coated samples present a high frequency capacitive loop and a diffusional response or a capacitive loop at a low frequency. The total impedance for both coatings is considerably larger than that for the uncoated surface, indicating that corrosion protection is being conferred by the coatings formed during conversion process. The form of the EIS response does not depend on immersion time in the coating bath. However, longer coating time results in a more protective coating, as evidenced by increased total impedances.

**Figure 6:**
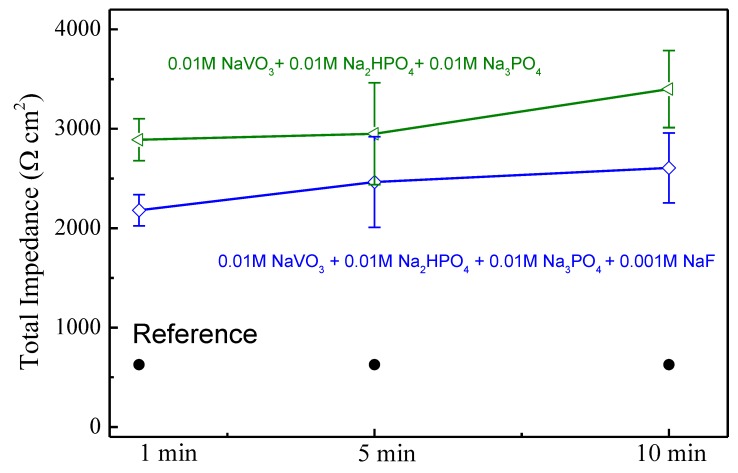
Total impedance of conversion coated AZ31.

Figure 6 shows the total impedance as a function of coating time for each of the coatings. The impedance of the coatings formed in the fluoride-free bath increase from 2.2 to 2.5 kΩ cm^2^, and those formed in the fluoride-containing bath increase from 2.9 to 3.4 kΩ cm^2^ as immersion time in the coating bath increases from 1 to 10 min. Both coatings present total impedances that are much greater than the uncoated AZ31 surface, whose impedance is about 1.0 kΩ cm^2^. The increases in impedance with increasing immersion time in the coating bath indicate that a protective film is forming [2,45]. However, the presence of fluoride in the coating bath has a negative effect, producing a coating whose total impedance is reduced by 20% to 25% compared to the fluoride-free conversion bath formulation.

## 4. Conclusions

The effect of millmolar additions of vanadate, phosphate, hydrophosphate and fluoride, and various pairings of these compounds, on the polarization response of AZ31 exposed to 0.1 M NaCl solution was examined. All of the compounds and their pairings reduced the corrosion rate of AZ31, except for fluoride. Varied effects on the breakdown and corrosion potential were observed.

1. The 10 mM Na_3_PO_4_–4 mM NaVO_3_ inhibitor pair is a powerful corrosion inhibitor for AZ31 during exposure to 0.1M NaCl solution, decreasing the corrosion rate by nearly two orders of magnitude compared to the corrosion rate in an uninhibited chloride solution. A robust and apparently protective surface film was observed in SEM images collected after exposure to the solution. An assessment of the corrosion rate data collected in this study, made according to the Bliss Independence model, indicates that this inhibitor pair acts synergistically under the conditions examined;

2. The 10 mM Na_2_HPO_4_–4 mM NaVO_3_ inhibitor pair was also effective in decreasing the corrosion rate of AZ31, and robust film formation was indicated. It decreases the likelihood of surface film breakdown. However, these compounds do not appear to act synergistically to improve corrosion rate. At longer exposure times, antagonism was indicated by the Bliss Independence assessment;

3. The 10 mM NaF–4 mM NaVO_3_ inhibitor pair was the least effective in terms of reducing the corrosion rate. There were indications of film formation in SEM images, but the Bliss Independence assessment indicated antagonism between these compounds;

4. Across all experiments, the presence of vanadate in solution was associated with the formation of a thin, but continuous surface film. Shrinkage cracking in the SEM vacuum suggests that the film is an amorphous gel in its as-formed state. The addition of phosphate to the solution leads to the formation of a thicker continuous film;

5. Concept conversion coatings were formed by immersion in a mixed phosphate–vanadate bath with and without fluoride. Coatings provided improved corrosion resistance during short term immersion in dilute chloride solutions. The present of 1 mM fluoride had the effect of degrading the coating protection by 20% to 25%.

## Figures and Tables

**Table 1:** The inhibitor content of each experiment.

No.	Inhibitor Content
1	0.1 M NaCl + 10 mM Na_3_PO_4_
2	0.1 M NaCl + 10 mM Na_2_HPO_4_
3	0.1 M NaCl + 10 mM NaF
4	0.1 M NaCl + 4 mM NaVO_3_ + 10 mM Na_3_PO_4_
5	0.1 M NaCl + 4 mM NaVO_3_ + 10 mM Na_2_HPO_4_
6	0.1 M NaCl + 4 mM NaVO_3_ + 10 mM NaF

**Table 2:** The “mixing effect” and Bliss Independence assessment of corrosion current density (i_corr_) for inhibitor pairs.

Inhibitors	i_corr_ Mixing Effect ^1^			i_corr_ Bliss Test		
Time	10 min	30 min	60 min	10 min	30 min	60 min
NaVO_3_ + Na_3_PO_4_	+	+	+	5.69	3.63	1.33
NaVO_3_ + Na_2_HPO_4_	+	+	−	1.24	0.47	0.14
NaVO_3_ + NaF	−	−	−	0.22	0.11	0.08

^1^ “+” represents positive mixing effect and “−” represents negative mixing effect.

**Table 3:** The “mixing effect” and Bliss Independence assessment of E_breakdown_-E_corr_ for inhibitor pairs.

Inhibitors	E_breakdown_-E_corr_ Mixing Effect ^1^			E_breakdown_-E_corr_ Bliss Test		
Time	10 min	30 min	60 min	10 min	30 min	60 min
NaVO_3_ + Na_3_PO_4_	−	−	−	0.68	0.78	0.89
NaVO_3_ + Na_2_HPO_4_	+	+	+	1.63	5.41	4.31
NaVO_3_ + NaF	+	+	+	1.58	1.82	1.49

^1^ “+” represents positive mixing effect and “−” represents negative mixing effect.
